# BA-CLM: A Globally Consistent 3D LiDAR Mapping Based on Bundle Adjustment Cost Factors

**DOI:** 10.3390/s24175554

**Published:** 2024-08-28

**Authors:** Bohan Shi, Wanbiao Lin, Wenlan Ouyang, Chenyu Shen, Siyang Sun, Yan Sun, Lei Sun

**Affiliations:** 1 Institute of Robotics & Automatic Information System, Nankai University, Tianjin 300350, China; 2120220539@mail.nankai.edu.cn (B.S.);; 2 Shenzhen Research Institute, Nankai University, Shenzhen 518081, China; 2120160381@mail.nankai.edu.cn

**Keywords:** consistent high-precision mapping, LiDAR bundle adjustment, graph optimization

## Abstract

Constructing a globally consistent high-precision map is essential for the application of mobile robots. Existing optimization-based mapping methods typically constrain robot states in pose space during the graph optimization process, without directly optimizing the structure of the scene, thereby causing the map to be inconsistent. To address the above issues, this paper presents a three-dimensional (3D) LiDAR mapping framework (i.e., BA-CLM) based on LiDAR bundle adjustment (LBA) cost factors. We propose a multivariate LBA cost factor, which is built from a multi-resolution voxel map, to uniformly constrain the robot poses within a submap. The framework proposed in this paper applies the LBA cost factors for both local and global map optimization. Experimental results on several public 3D LiDAR datasets and a self-collected 32-line LiDAR dataset demonstrate that the proposed method achieves accurate trajectory estimation and consistent mapping.

## 1. Introduction

Simultaneous localization and mapping (SLAM) is widely used in the field of robotics. A consistent map is crucial for mobile robots to plan and navigate. It is desirable for a mapping method to have the capacity to construct a globally consistent high-precision map, especially in large-scale scenes.

SLAM can be divided into filter-based methods and optimization-based methods. While state-of-the-art filter-based SLAM methods [1,2,3] can achieve high precision and robust positioning in the short term, they cannot correct the errors in the previous states and are therefore not suitable for globally consistent mapping. For the sake of efficiency, optimization-based SLAM approaches solve the pose graph optimization problem to construct a global map. The pose graph constrains states based on relative poses, which are commonly obtained through scan matching. However, accurately modeling the noise for relative poses is difficult in practice, which hinders the effective guarantee of map consistency.

Constraining states based on the matching cost is another approach used to achieve globally consistent mapping for optimization-based SLAM. D. Borrmann et al. [4] solved the matching cost minimization problem on a global scale after each pose graph optimization process to ensure the global consistency. K. Koide et al. [5] proposed a GPU-accelerated generalized iterative closest point (GICP) [6] factor to achieve the real-time solving of the global matching cost minimization problem in the factor graph for globally consistent map construction. However, these approaches are limited by the shortcomings of iterative closest point (ICP)-based matching algorithms, as they only align every two frames of point clouds instead of directly optimizing the structure of the entire local map. This limitation results in drawbacks in terms of accuracy.

To address the aforementioned issues, a LiDAR bundle adjustment (LBA) cost factor [7] is proposed for map optimization in this paper. The proposed factor calculates the LBA cost using a multiple-frame synthesized voxel map and constrains the robot states through the matching cost. The proposed factor offers several advantages. First, the multivariate factor can uniformly constrain all states within a submap, which makes the constraints on the robot’s states tighter and more consistent compared with the traditional binary factor. Second, the synthesized voxel map effectively addresses the sparse nature of low-resolution LiDAR and is more efficient than k-d-tree-based maps. Finally, the LBA factor can be integrated with factors derived from measurements of other sensors for multi-sensor fusion mapping.

Based on the proposed LBA cost factor, we further introduce BA-CLM: a globally consistent LiDAR mapping framework that incorporates LBA refinement into factor graph optimization for both local and global mapping. Unlike other current methods, BA-CLM consistently optimizes poses to minimize global LBA costs in real time, resulting in accurate pose estimation and high-quality map construction, even in challenging long-term mapping scenarios. The framework of BA-CLM consists of three modules: front-end odometry, local mapping, and global mapping. A sliding window approach is involved in the local mapping module to create LBA cost factors for the frames within the window, which are then added to the local factor graph to perform the local map optimization. Based on the overlap with previous keyframes, the latest marginalized frame will be filtered as a new keyframe. In the global mapping module, GPS absolute measurements and Scan context [8] (a fast encoding-based loop closure detection method) are introduced to effectively eliminate accumulated errors. The LBA cost factors are created for the keyframes within the detected loop to achieve globally consistent map optimization.

**Figure 1:**
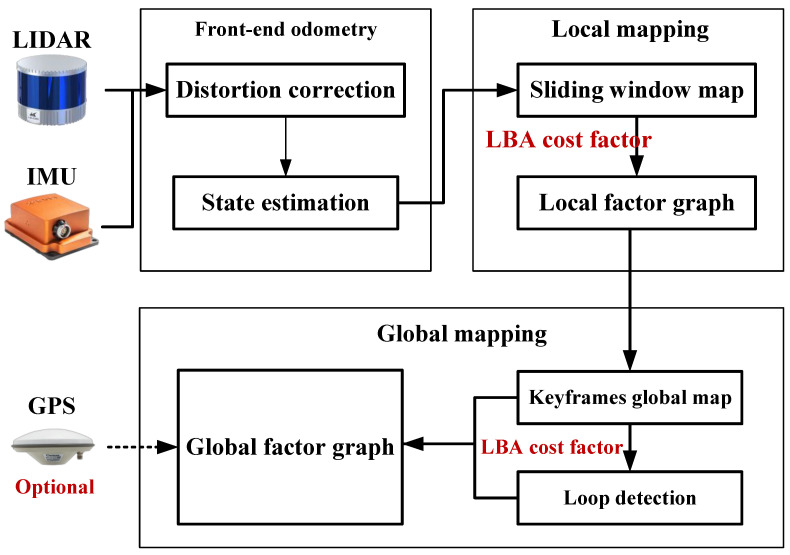
Block diagram of the proposed framework.

The main contributions of this paper can be summarized as follows:

- A voxel-based multivariate LBA cost factor is proposed for consistent mapping, which is created from a synthesized multi-resolution voxel map.
- A real-time globally consistent 3D LiDAR mapping framework (see Figure 1) is presented based on the LBA cost factor and CPU parallel computing.
- The efficiency and effectiveness of the proposed work are extensively validated on multiple public and self-collected LiDAR datasets.

## 2. Related Work

### 2.1. Graph-Based LiDAR SLAM

The LiDAR SLAM problem can be represented as a graph [9] that consists of nodes (poses) and edges (constraints) and can be solved with optimization methods (e.g., the Levenberg–Marquardt algorithm [10]). The iSAM proposed in [11] introduces incremental updates to the graph optimization process, enabling graph-based SLAM systems for real-time applications. For large-scale and long-term mapping, the graph-based SLAM adopts the method of global optimization and closed-loop processing, which can be used to improve the quality of the map.

A pose graph is a kind of graph that mainly imposes pose constraints. There are extensive LiDAR SLAM studies [12,13,14] that rely on pose graph optimization as the back-end to refine the trajectory estimation results. LIO-SAM [15] introduces the concept of a keyframe to back-end optimization, which significantly reduces the dimensionality of the pose graph. MULLS [16] proposes a two-stage optimization strategy for global pose graph optimization and further improves the convergence speed in the loop closure condition. The aforementioned approaches make pose-graph-based SLAM a low-computation-cost method. However, the traditional pose-graph-based approaches have many deficiencies. First, it is a general solution for pose-graph-based SLAM to roughly approximate the uncertainty of the pose constraint as a constant, which is difficult to evaluate in practice, leading to inaccuracy in the estimation. Second, the iterative solution process may easily converge to a local solution, particularly for the three degrees of freedom of rotation. Finally, the pose constraints fail to consider the refinement of the scene structures, causing the map to lose consistency.

To obtain an accurate and consistent map, matching costs can be directly added to the graph as constraints on the poses. In the series works by K. Koide et al. [5,17], voxelized GICP matching cost factors were added to the factor graph to minimize the global registration error over the entire map. However, this approach is not robust to the sparse nature of LiDAR scans for calculating the matching cost only between two frames of point clouds in the GICP method. Furthermore, the point cloud registration methods simply align the nearest feature points without considering the distribution of features, which may disrupt the overall structure of the features. Based on the above analysis, acquiring the matching cost using the structure-from-motion method is a more reasonable choice than acquiring it from a point cloud registration method, because a structure-from-motion method can efficiently refine the scene structure at the same time. Therefore, we propose an LBA cost factor in this paper, which uniformly and compactly constrains the states within a large range for accurate globally consistent mapping.

### 2.2. LiDAR Bundle Adjustment

Bundle adjustment (BA), which simultaneously optimizes the robot poses and scene structure by minimizing the reprojection errors of the matched features, is widely used in the vision-based structure-from-motion and SLAM methods [18,19,20]. In LiDAR mapping, LBA differs significantly from traditional vision-based BA, as the sparse nature of point clouds makes exact feature matching impossible. BALM [7] first formulates the LBA by minimizing the distance between a feature point and its matched feature. BALM2 [21], a subsequent version of BALM, derives closed-form derivatives for LBA optimization and develops an efficient second-order LBA solver based on the concept of point clusters. BA-LIOM [22] addresses the issue of ground warping utilizing an LBA solver to provide pose estimations for the pose-graph-based back-end. Given a good initial pose estimation, HBA [23] improves the mapping quality by employing a two-step approach involving bottom-up hierarchical BA and top-down pose graph optimization. While the offline method HBA demonstrates the potential of LBA in reconstructing accurate and consistent large-scale LiDAR point cloud maps, the high computational costs limit the application of global LBA optimization for real-time consistent mapping systems.

### 2.3. Voxel Map

A voxel map is a hash-based map structure proposed in [24]. Compared with a k-d tree, a map structure that widely used for point cloud registration and SLAM, a voxel map can be built and updated more efficiently. A modified voxel method, iVox, was proposed in [25], which supports incremental insertion and parallel approximated k-nearest neighbor (k-NN) queries and further improves the search and update efficiency of the voxel map. VoxelMap++, presented in [26], uses a union-find-based voxel merging method to enhance the accuracy of plane fitting. K. Koide et al. [27] proposed a multi-point distribution aggregation approach to robustly estimate the distribution of a voxel and released an open source voxel project that can be rapidly constructed with parallel computation.

Previous LBA studies did not focus much on improvements in computation speed, which limits the applicability of LBA methods in global mapping. Traditional LBA approaches [21,23] use an octree-based voxel map method to segment local LiDAR scans into point clusters. This octree-based map method requires eigenvalues to be computed for each voxel during the voxel cutting process, resulting in extremely high computational costs that make real-time solutions for large-scale LBA problems impractical. However, through improving the map structure, eigenvalue calculations can be separated from the voxel cutting process. This separation allows the numerous repetitive calculations to be efficiently managed through parallel computing. Therefore, this paper introduces a multi-resolution voxel map with parallel computing acceleration to address the issue of computation speed in LBA.

## 3. Methodology

### 3.1. LiDAR Bundle Adjustment Cost Factor

Given *M* frames of point clouds $F=\left\{f_{1},\cdots ,f_{M}\}\right.$, the robot poses corresponding to the frames can be written as

(1) $$\begin{array}{c} T=\left\{T_{f_{1}},\cdots ,T_{f_{M}}\}\right., \end{array}$$

where $T_{f_{m}}\in SE\left(3\right)$ is the transformation matrix from the body to the world. These point clouds are aligned to the world coordinate system and can be segmented into a multi-resolution voxel map. The number of voxels is *C*. The full voxel map can be denoted as $V=\left\{v_{1},\cdots ,v_{C}\}\right.$. All the points within the i-th voxel $P_{v_{i}}^{w}=\left\{p_{v_{i1}}^{w},\cdots ,p_{v_{iN}}^{w}\right\}$ constitute a point cluster that corresponds to a planar feature. The distribution covariance matrix of $v_{i}$ is

(2) $$A_{v_{i}}=\frac{1}{N}\sum_{j=1}^{N}\left(p_{v_{ij}}^{w}-{\bar{p}}_{v_{i}}^{w}\right)\in R^{3\times 3},$$

where $p_{v_{ij}}^{w}$ and ${\bar{p}}_{v_{i}}^{w}$ are

(3) $$p_{v_{ij}}^{w}=R_{f_{m}}p_{v_{ij}}^{f_{m}}+t_{f_{m}},$$

(4) $${\bar{p}}_{v_{i}}^{w}=\frac{1}{N}\sum_{j=1}^{N}\left(p_{v_{ij}}^{w}\right).$$

The eigenvalues of $A_{v_{i}}$ describe the distribution of points within the voxel. According to principal component analysis (PCA) theory [28], the eigenvalues’ cumulative contribution to $A_{v_{i}}$ is calculated to judge whether the voxel corresponds to a planar feature. In this paper, we discriminate the features based on the following equation:

(5) $$Feature\left(v_{i}\right)=\left\{\begin{array}{cc} plane & \mathrm{if}\;\frac{\lambda_{1}+\lambda_{2}}{\lambda_{1}+\lambda_{2}+\lambda_{3}}\geq \theta \\ nonfeature & \mathrm{else} \end{array}\right.,$$

where θ is a constant threshold (e.g., 0.9), and $\lambda_{k}$ is the k-th largest eigenvalue of $A_{v_{i}}$.

Although the points within the extracted voxel according to (5) are distributed in a plane as a whole, there is still a possibility of having outliers. Due to the interference of measurement errors and noise, the distance from the planar feature points to the plane should follow a normal distribution with a mean of zero, that is,

(6) $$d_{p_{vij}}=\left(p_{v_{ij}}^{w}-{\bar{p}}_{v_{i}}^{w}\right)\cdot {\overset{⇀}{\;a}}_{3}∼N(0,\lambda_{3}),$$

where ${\overset{⇀}{\;a}}_{3}$ is the eigenvector of λ_3_ and also a unit vector in the feature plane normal direction. Hence, the outliers are removed according to the three-sigma rule of thumb.

The goal of LBA optimization is to estimate the optimal robot pose by minimizing the sum of the smallest eigenvalues of the covariance matrices of all voxels. For one voxel, the LBA error is

(7) $$e_{v_{i}}^{LBA}\left(T,p\right)=\lambda_{3}\left(A_{vi}\right)=u_{3}^{T}A_{vi}u_{3},$$

where $u_{3}$ is the corresponding eigen vector of $\lambda_{3}$. For a local map, the entire LBA error and LBA optimization problem can be defined as follows:

(8) $$e^{LBA}\left(T,p\right)=\sum_{i=1}^{C}e_{v_{i}}^{LBA},$$

(9) $$T^{*}=\arg\min_{T}e^{LBA}\left(T,p\right).$$

The LBA optimization problem is rewritten with a Hessian factor in the form of GTSAM (i.e., LBA cost factor). The matrix used to construct the LBA cost factor is shown as follows [7]:

(10) $$f=2e^{LBA}\left(T,p\right)\in R^{1},$$

(11) $$\begin{array}{cc} g_{m} & =-\frac{\partial e^{LBA}}{\partial T_{fm}}\\ \; & =-\sum_{v_{i}\in V}{\sum_{p\in v_{i}\cap f_{m}}\frac{2}{N}\left(p^{w}-{\bar{p}}_{v_{i}}^{w}\right)}^{T}{u_{3}}^{T}u_{3}\left[\begin{array}{cc} -R_{m}^{w}{\left(p^{f_{m}}\right)}^{\Lambda } & I \end{array}\right]\in R^{6\times 1} \end{array}$$

(12) $$G_{mn}=-2\frac{\partial g_{m}}{\partial T_{fn}^{w}}\in R^{6\times 6},$$

(13) $$M_{hessian}=\left(\begin{array}{ccccc} G_{11} & G_{12} & \cdots & G_{1M} & g_{1}\\ 0 & G_{22} & \cdots & G_{2M} & g_{2}\\ \vdots & \vdots & \ddots & \vdots & \vdots \\ 0 & 0 & 0 & G_{MM} & g_{M}\\ 0 & 0 & 0 & 0 & f \end{array}\right).$$

**Figure 2:**
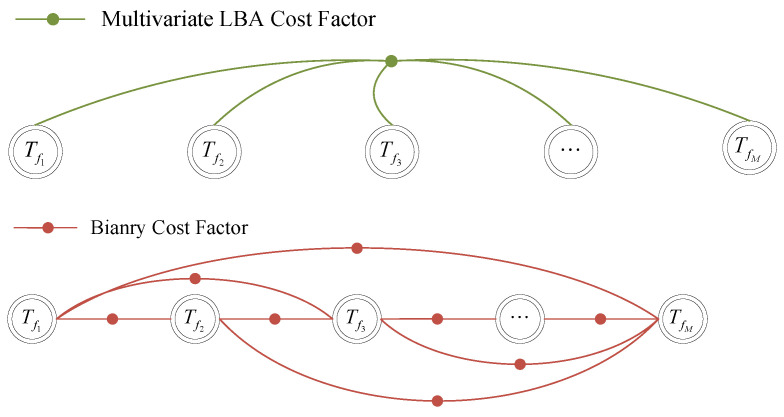
For the same submap, the robot poses can be constrained by one proposed LBA cost factor or a substantial number of traditional binary cost factors in a fully connected form.

The diagram of the LBA cost factor is shown in Figure 2. The matching cost is calculated from the entire voxel map, rather than from a pair of point clouds. Thus, compared with binary factors, the proposed factor effectively solves the general problem of ground curling and is more robust to the sparsity of point clouds.

### 3.2. Multi-Resolution Voxel Map

To address the difference in feature scales, [7] proposes an adaptive octree-based voxelization method. This method is extremely time-consuming for global mapping because it needs to calculate the eigenvalues of every voxel and iteratively subdivide each default-sized voxel until all the subdivided points belong to the same feature or reach the maximum level of subdivision. To cut the point cloud into point clusters in real time, we abandoned the octree data structure in our research. Inspired by [29], a multi-frame synthesized voxel pyramid method is adopted to extract the planar point clusters at different scales, which can be rapidly constructed through parallel computing.

Considering the sparse nature of point clouds, we accumulate a certain number of point clouds to form a submap. Each submap corresponds to an LBA cost factor. For a submap, we simultaneously voxelize all the frames into three layers. During the optimization process, we extract all the voxels that store planar features via (5) from the lowest-resolution layer to the highest-resolution layer. All the extracted voxels are used to construct the LBA cost factor of the map. To avoid redundant constraints, the extracted voxels in the lower layer will be disabled in the upper layer. When a frame is removed from the submap, all planar points are retained to prevent submap drifting.

Despite its higher computational complexity, the multi-resolution method proposed in this paper significantly reduces the time complexity compared with the method proposed in [7], as it can be implemented through parallel computing.

### 3.3. Global Mapping Framework

The proposed framework is shown in Figure 1. It is worth noting that the LBA cost factor relies on relatively accurate voxel segmentation. Thus, when the new LiDAR frame comes in, a front-end odometry module is required to provide an initial guess for the robot pose of the frame. In this work, we use Faster-LIO [25], a voxel-based lightweight LiDAR odometry module, for point cloud distortion correction and initial state estimation. This module can be replaced by any LiDAR odometry or point cloud registration algorithm, such as TEASER [30] and NDT [31].

#### 3.3.1. Local Mapping Module

**Figure 3:**
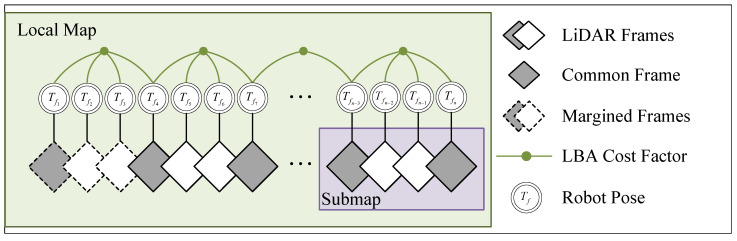
Factor graph for the local mapping module. For the purpose of demonstration, the submap size in this figure is 4, and the step length is 3. For this work, we set the maximum number of factors to 8, the submap size to 10, and the step size to 5.

The LiDAR frame with an initial guess is fed into the local mapping module to obtain a more accurate pose estimate, which is summarized in Algorithm 1. The local mapping module manages the frames via a sliding window. The accumulated frames are synthesized and constructed into a multi-resolution voxel submap with a certain step length. To avoid drifting between submaps, some frames (i.e., common frames) are inserted into both of the adjacent submaps to keep an overlap area between them (similar to HBA [23]). Due to the presence of common frames, the new submap can be incrementally constructed based on the previous submap through pop and push operations. This incremental method is much more rapid and efficient than constructing a brand new voxel map. We construct LBA cost factors for all submaps in the local map and feed them into the local factor graph (see Figure 3) for optimization. After each optimization, the positions of points within the map will be updated. The voxel submap will only be recut when the robot pose increment is greater than 50% of the lowest-layer voxel edge length. After local map optimization, the frames in the oldest submap are marginalized, which means that the relative transformations among them are fixed.
**Algorithm 1:** Local Mapping
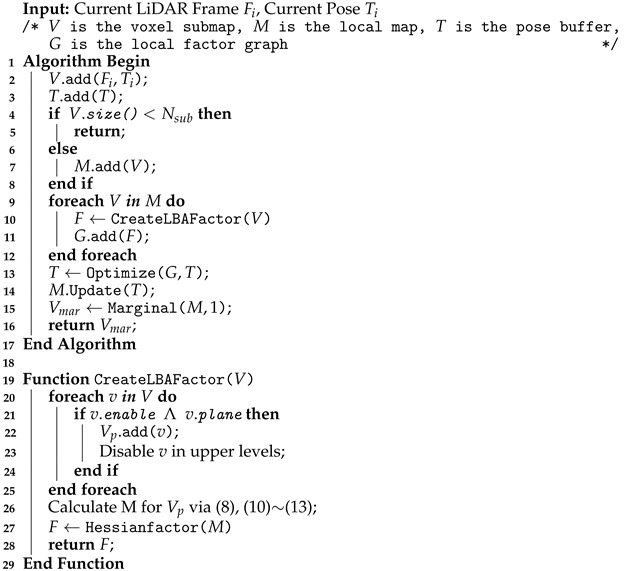


#### 3.3.2. Global Mapping Module

The significance of the global mapping module is to maintain the consistency of the global map in long-term mapping. To speed up global map optimization, we reduce the number of pose variables by merging the frames of the same submap into one. As mentioned in Section 3.2, the points from previous frames are preserved in the new marginalized submap. We define the overlap ratio of the new marginalized submap as

(14) $$s\left(v_{i}\right)=\left\{\begin{array}{cc} 1 & \mathrm{if}\;v_{i}\;\mathrm{has}\;\mathrm{more}\;\mathrm{than}\;p\;\mathrm{previous}\;\mathrm{points}\\ 0 & \mathrm{else} \end{array}\right.,$$

(15) $$ratio\left(V\right)=\frac{\sum_{i=1}^{C}s\left(v_{c}\right)}{C}\times 100\%,$$

where *p* is a constant related to the number of LiDAR lines and the step length. In this work, the submap merging frame becomes a new keyframe if the ratio given by Equation (15) is less than 80%. Initially, we align the trajectory of the local mapping and GPS to calibrate the transformation between the world coordinate system and the ENU coordinate system. Following calibration, GPS factors are periodically added to the global factor graph to constrain the pose of keyframes. We utilize Scan context [8] to detect the closed-loops among keyframes. Once a closed-loop is detected, LBA cost factors are generated for all keyframes within the shortest closed-loop, which makes it possible to perform BA optimization on frames that are spatially close but have significant time intervals. This process refines the map structure for a large region, but it is time consuming. Therefore, global map optimization will take place at large time intervals.

### 3.4. Implementation Detail

Both CPU and GPU parallel acceleration can be applied to the improved voxel map. In this work, we used the OpenMP library to implement CPU parallel computing acceleration. We assigned voxels to threads to create, update, recut, and calculate the LBA cost for the voxel map. Through CPU parallel computing, BA-CLM can run in real time (see Section 4.3).

## 4. Evaluation

### 4.1. Public Datasets

**Figure 4:**
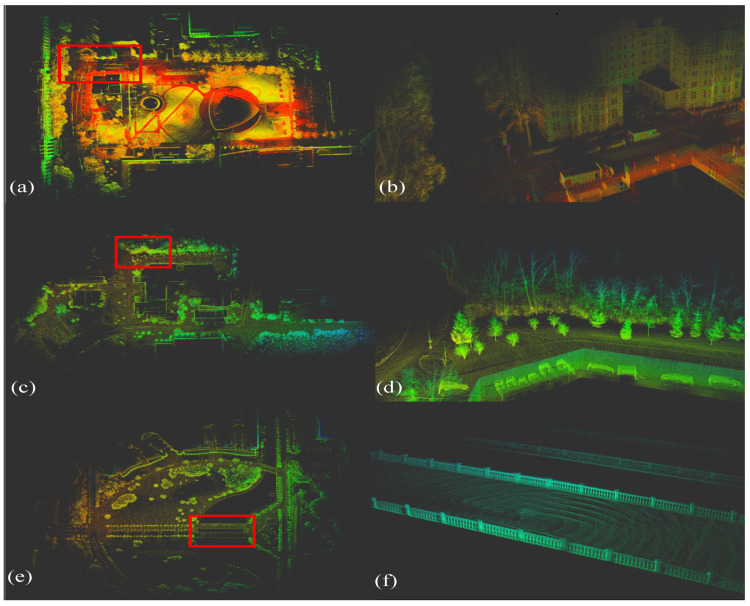
Detailed mapping result of BA-CLM on public datasets, (**a**,**b**) LIO-SAM-WEST, (**c**,**d**) NCLT-20130110, (**e**,**f**) M2DGR-STREET04. The first column of images displays the bird’s eye views of the overall scenes. The second column shows the corresponding details of the map locations highlighted by red boxes.

To evaluate the accuracy, BA-CLM was tested on the KITTI [32], M2DGR [33], and NCLT [34] datasets (see Figure 4), with the root mean square error (RMSE) of the absolute trajectory error (ATE) serving as the accuracy evaluation metric. The LiDAR data were collected from a 64-line LiDAR system for KITTI and from a 32-line LiDAR system for M2DGR and NCLT.

#### 4.1.1. KITTI and M2DGR

On these two datasets, BA-CLM was compared with several state-of-the-art real-time 3D LiDAR SLAM methods: Faster-LIO [25], FAST-LIO2 [1], LIO-SAM [15], BALM2 [21], HBA [23], A-LOAM (an improved version of LOAM [35]), and PIN-SLAM [36]. To ensure the rigor of the experiment, GPS measurements were prohibited from being provided to BA-CLM. Additionally, the motion compensated point clouds with initial pose estimates for the BALM2, HBA, and PIN-SLAM came from Faster-LIO (same as for BA-CLM), rather than from MULLS [16]. The HBA reached the optimal outcome within five iterations of global BA, which means that the runtime for the HBA was close to the duration of the sequences. Therefore, the experimental results in this study were different from those in [23,36].

Experimental results on the partial sequences are reported in Table 1. These show that BA-CLM yielded the highest or second-highest accuracy on most sequences. On the sequences without a loop (marked with ‘*’), the global mapping module of BA-CLM was deactivated. The ATE of the local mapping module of BA-CLM was still equivalent to that of the global BA method HBA and much lower than those of the other methods. On the sequences with loops, BA-CLM exhibited extremely high accuracy. The increase in accuracy was caused by the added LBA cost factors created by loop detection. Notably, there was one exception: BA-CLM had a lightly larger ATE on KITTI07. In the sequence, a large initial error led to an incorrect construction for the LBA cost factor. This confirmed the dependence of the LBA method on relatively exact initial estimates. However, this issue could be efficiently solved by limiting the trajectory errors to a range through the addition of GPS absolute measurements.

**Figure 5:**
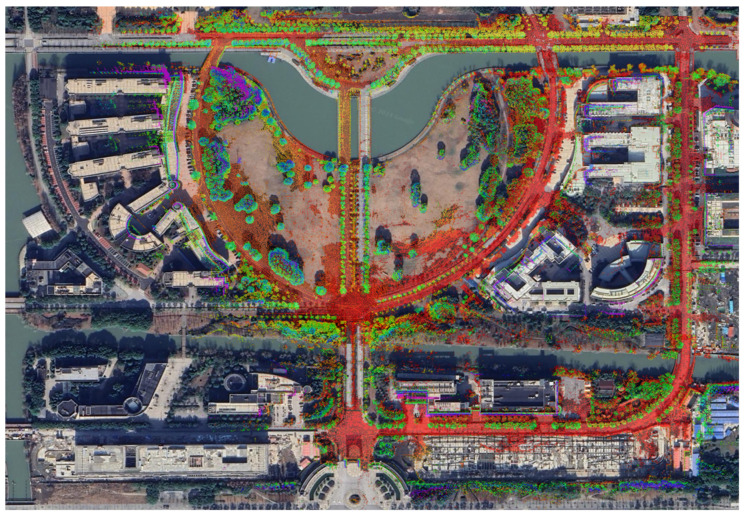
Mapping result of BA-CLM aligned with Google earth on M2DGR dataset [33].

The Mapping result of BA-CLM on M2DGR dataset is shown in Figure 5. Compared to the other two LBA-based methods (BALM2 [21] and HBA [23]), BA-CLM demonstrates the highest overall accuracy. BALM2 employs a single sliding window for mapping, which significantly limits the scale of LBA refinement. Although HBA performs global LBA refinement across all frames, its subsequent pose graph optimization process may increase LBA costs, potentially causing the refinement to converge more slowly or become trapped in a local minimum. In contrast, BA-CLM is based on the matching cost minimization method, which consistently determines the optimal poses to minimize LBA costs. Moreover, once a loop is detected, BA-CLM constructs LBA cost factors for keyframes that are spatially close, further enhancing accuracy, whereas HBA only performs LBA refinement for temporally close frames.

#### 4.1.2. NCLT

The NCLT dataset [34], which has a much longer duration than the KITTI and M2DGR datasets, was adopted to evaluate the accuracy of BA-CLM for long-term mapping.

**Figure 6:**
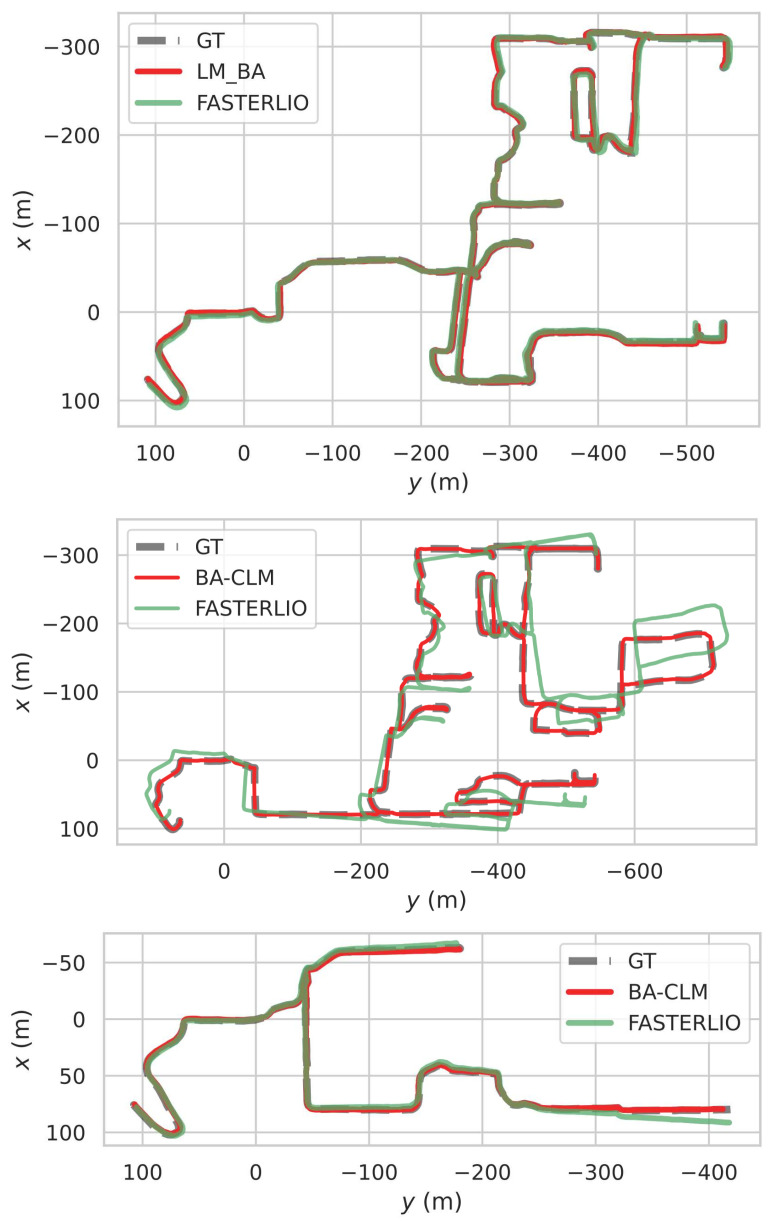
Trajectories of BA-CLM on sequence 20120429, 20120615, and 20130110 of the NCLT dataset [34]. These show that BA-CLM achieved accurate trajectory estimation despite the initial guess deviating from the ground truth.

The RMSEs of the ATE on the NCLT dataset are summarized in Table 2. The duration of the sequences listed was 2597.130, 5072.362, 3309.720, and 1021.680 s. As can be seen, despite Faster-LIO [25] (the initial guess for BA-CLM) having a high average error (0.476%), BA-CLM still showed great accuracy in trajectory estimation (the average error was 0.052%). The trajectories of BA-CLM on sequences 20120429, 20120615, and 20130110 are shown in Figure 6. It is evident that BA-CLM successfully corrected the trajectories, regardless of whether Faster-LIO significantly deviated from the ground truth. This demonstrates that BA-CLM only needs a relatively accurate relative pose as an initial guess to achieve accurate estimation over a global range, even in long-term operating situations.

### 4.2. Self-Collected Dataset

The focus of this discussion is the map consistency. BA-CLM was compared with FAST-LIO2 (an odometry method without a back-end) and LIO-SAM (a pose-graph-based mapping method) on a mid-term self-collected dataset (the trajectory length ranged from 1000 to 2000 m). The sensors we used for this dataset included a LSLIDAR 32-line LiDAR, a nine-axis inertial measurement unit (IMU), and a real-time kinematic positioning (RTK) system. During the collection process, a vehicle was equipped with the aforementioned sensors and driven in an urban environment at a speed of around 30 km/h.

**Figure 7:**
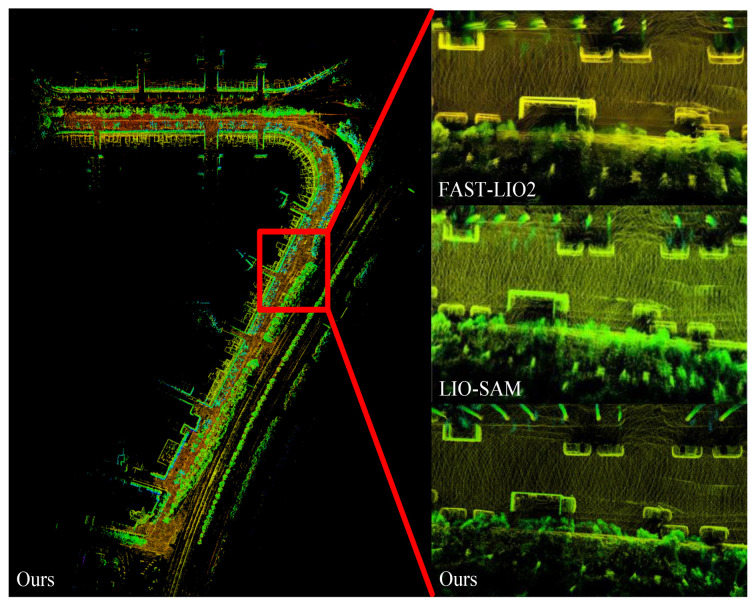
Mapping result of BA-CLM on the self-collected dataset presented from a bird’s eye view. The partial map highlighted by the red boxes is zoomed in on and compared with the FAST-LIO2 and LIO-SAM results [1,15].

The mean map entropy (MME [37]) was adopted to evaluate the mapping consistency. We built a submap every 10 m and calculated the average MMEs of all the submaps. The average MMEs on the self-collected dataset are shown in Table 3. BA-CLM showed the smallest MMEs (−1.767, −1.824, and −2.113) on all the three sequences, while they exhibited larger MMEs for FAST-LIO2 and LIO-SAM. Figure 7 shows the mapping result. The map constructed by BA-CLM maintained good quality. Meanwhile, FAST-LIO2 exhibited significant cumulative errors and severe stratification on the ground and walls. LIO-SAM correctly detected the loop and eliminated the cumulative errors, but the trajectory was not completely corrected due to the influence of the previous odometry factors. This showed that the pose-graph-based method only optimized in pose space without considering the map consistency, resulting in map divergence. In conclusion, thanks to the matching cost minimization approach, BA-CLM has a strong capacity to maintain map consistency.

### 4.3. Runtime Analysis

Real-time performance is an important specification in SLAM systems. We tested the efficiency of BA-CLM on several 32-line LiDAR datasets. The computation platform used for the tests was a laptop equipped with an Intel i9-12900H 2.50 GHz CPU and 32 GB of RAM.

**Figure 8:**
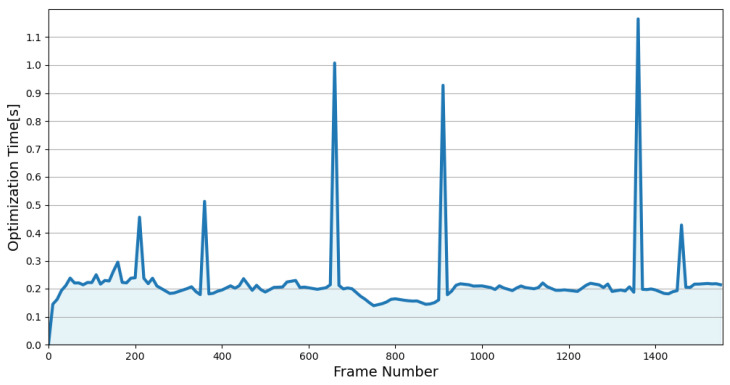
The runtime of BA-CLM on sequence 02 of the self-collected dataset. The peaks correspond to the global map optimization.

The detailed runtime of BA-CLM is summarized in Table 4. The first line presents the time cost for placing points into their corresponding default-sized voxels. The average time required for this step was reduced from 58.7 ms to 16.8 ms through incremental construction. The second line indicates the time taken to construct multi-resolution voxel maps for all submaps, which was executed at a frequency of twice per second. It took 542.5 ms (67.8 ms per submap) to construct the maps under single-thread conditions (using the method proposed in [21]), while BA-CLM completed the multi-resolution map construction in only 37.2 ms under CPU parallel computing conditions. The local map optimization, executed at a frequency of once per second, required an average of only 217.1 ms. The global map optimization performed the BA refinement on a much larger scale than the local optimization, resulting in an average time consumption of 847.2 ms. However, it was only executed when a closed-loop was detected, which means there was an interval of a few minutes between the two global optimization processes (as can be seen in Figure 8). Overall, the average processing time per frame of BA-CLM (55.3 ms) was much lower than the LiDAR scan interval (100 ms). We can conclude that BA-CLM could run in real time.

## 5. Conclusions

In this paper, we propose a uniform and tight multivariate LBA cost factor for global mapping to improve the accuracy of pose estimation and the consistency of the map. A multi-resolution voxel map that supports both CPU and GPU parallel acceleration is used to rapidly create the LBA cost factor. We present a real-time global mapping framework based on the proposed factor, which minimizes the matching cost via factor graph optimization. Abundant experiments on several datasets demonstrated that BA-CLM outperforms other state-of-the-art SLAM methods in terms of accuracy. In future work, we will fuse more kinds of raw sensor measurements (e.g., wheel speed sensor, IMU, and 4D radar) into our framework to improve the robustness.

## Figures and Tables

**Table 1:** Root mean square errors (RMSEs) of absolute trajectory error (ATE) [***m***] on public datasets.

Sequence	BA-CLM	Faster-LIO	FAST-LIO2	LIO-SAM	BALM2	HBA	A-LOAM	PIN-SLAM
M2DGR-S02 *	1.823	2.666	2.323	4.063	2.012	1.742	5.299	2.081
M2DGR-S03	0.118	0.498	0.198	0.192	0.249	0.143	0.586	0.138
M2DGR-S04	0.225	1.182	0.443	1.022	0.525	0.439	2.337	0.454
M2DGR-S05 *	0.295	1.182	0.378	1.022	0.601	0.278	1.364	0.581
M2DGR-S06 *	0.384	0.457	0.413	0.417	0.397	0.400	0.682	0.777
M2DGR-S07 *	10.772	11.736	11.751	28.642	11.851	9.899	28.940	16.881
KITTI00	1.227	4.602	3.851	8.017	2.732	1.557	19.417	1.588
KITTI04 *	0.361	0.752	0.555	0.743	0.633	0.565	0.593	0.326
KITTI06	0.272	1.044	1.296	0.872	0.677	0.532	1.189	0.434
KITTI07	0.935	1.456	0.883	0.639	0.623	0.548	1.301	0.419

* The optimal results are marked in red, and the suboptimal results are marked in blue.

**Table 2:** RMSEs of ATE [***m***] on the NCLT dataset.

Sequence	Length [m]	BA-CLM	Faster-LIO
20120429	3186.047	**1.268**	5.882
20120511	6120.648	**2.299**	47.682
20120615	4088.233	**1.863**	25.737
20130110	1137.320	**0.966**	3.560
average [%]		**0.052%**	0.476%

The best results are highlighted in bold.

**Table 3:** Average mean map entropy (MME) (r = 0.3***m***) on the self-collected dataset.

Sequence	Length [*m*]	BA-CLM	FAST-LIO2	LIO-SAM
01	1470.785	**−1.767**	−1.412	−1.472
02	1095.689	**−1.824**	−1.506	−1.679
03	1872.245	**−2.113**	−1.712	−1.860

The best results are highlighted in bold.

**Table 4:** Runtime of BA-CLM on 32-line LiDAR datasets.

Step	Time Cost [ms]
Point Cloud Voxelization	58.7±10.9*
	16.8±4.8
Multi-resolution Voxel Map Construction	542.4±217.9*
	37.2±4.4
Local Map Optimization	217.1.0±29.5
Global Map Optimization	847.2±240.32
Average Time Per Frame	55.3±14.7

The time costs marked with * are without incremental construction and parallel acceleration.

## Data Availability

Data are contained within the article.
